# The Great East Japan Earthquake Disaster: a Compilation of Published Literature on Health Needs and Relief Activities, March 2011-September 2012

**DOI:** 10.1371/currents.dis.771beae7d8f41c31cd91e765678c005d

**Published:** 2013-05-13

**Authors:** Sae Ochi, Virginia Murray, Susan Hodgson

**Affiliations:** MRC-HPA Centre for Environment and Health, Imperial College London, London, United Kindom; Health Protection Agency; School of Public Health, Imperial College London, London, United Kingdon

## Abstract

Objective To provide an overview of the health needs following the Great East Japan Earthquake Disaster and the lessons identified. Methods The relevant of peer review and grey literature articles in English and Japanese, and books in Japanese, published from March 2011 to September 2012 were searched. Medline, Embase, PsycINFO, and HMIC were searched for journal articles in English, CiNii for those in Japanese, and Amazon.co.jp. for books. Descriptions of the health needs at the time of the disaster were identified using search terms and relevant articles were reviewed. Findings 85 English articles, 246 Japanese articles and 13 books were identified, the majority of which were experience/activity reports. Regarding health care needs, chronic conditions such as hypertension and diabetes were reported to be the greatest burden from the early stages of the disaster. Loss of medication and medical records appeared to worsen the situation. Many sub-acute symptoms were attributed to the contaminated sludge of the tsunamis and the poor living environment at the evacuation centres. Particularly vulnerable groups were identified as the elderly, those with mental health illnesses and the disabled. Although the response of the rescue activities was prompt, it sometimes failed to meet the on-site needs due to the lack of communication and coordination. Conclusion The lessons identified from this mega-disaster highlighted the specific health needs of the vulnerable populations, particularly the elderly and those with non-communicable diseases. Further research is needed so that the lessons identified can be incorporated into future contingency plans in Japan and elsewhere.

## Introduction

Effective disaster preparedness can be achieved by taking a comprehensive and panoramic view of a disaster. So far no paper has reviewed an overall health impact neither from an urban disaster nor from an earthquake.

The Great East Japan Earthquake Disaster (GEJED) in 2011 was one of the greatest natural disasters that occurred in modern society. An earthquake with a magnitude 9.0 on the Richter scale and the subsequent five to six tsunamis, reaching up to 38 m from sea level and flooded 561 km^2^ of the coastal area, killed more than 15,000 people in Japan.^1^ The GEJED was different from past earthquakes in many ways. Firstly, the unprecedented size of both the earthquake and the associated tsunamis were beyond the scope of even the most recent regulatory and operational projections. For example, the Fukushima Nuclear Power Plant to were prepared for an earthquake with magnitude 7.6,^51^ which turned out to be insufficient in this case. Secondly, this disaster had the distinct features of an urban disaster, typified by extensive power and water supply failures. Thirdly, recovery of infrastructure took much longer than anticipated in the current disaster management platform in Japan.^3^ Finally, the areas most affected were cities with a predominantly elderly population (30% above 60 years-old).^1^

The aim of this research is to understand what happens when a mega-disaster hit modern society. To achieve this objective, this paper reviews the available peer reviewed and ‘grey literature’ publications between March 2011 and September 2012, and summarises the health impacts at the time of the GEJED. Although the effect of the earthquake and the tsunamis impacted on the accident at the Fukushima-Daiichi nuclear power plant, this is specifically excluded in this literature review. This is because the health needs and health impacts related to the radiation exposure is very different from those related to the earthquake and tsunamis.

## Methods

Publicly available information written in English or Japanese was obtained from the following sources. The relevant literature was analysed and descriptions of the health needs and relief activities at the time of the GEJED were identified. As stated above, because health needs/impact related to radiation exposure is different from earthquake and tsunamis, articles mainly focus on nuclear issues were excluded in this review.

**Identification**

**1) Academic and ‘grey literature’ English journals**

The key health journal databases (Medline, Embase, PsycINFO, and Health Management Information Consortium (HMIC)) were searched via OvidSP. As many different names are used for the GEJED in English articles, the keywords used were ‘earthquake’ [AND] ‘Japan’ [AND] ‘health’ in all fields; ‘earthquake’ [AND] ‘Japan’ [AND] ‘hospital’ in all fields; or ‘earthquake’ [AND] ‘Japan’ [AND] ‘medicine’ in all fields.

**2) **Academic and ‘grey literature’ journals and books in Japanese****

The literatures written in Japanese are often searchable in Japanese words. Therefore, the review was conducted on the database provided by the National Diet Library in Japan (*CiNii*), using search term ‘東日本大震災 (GEJED)’ [AND] ‘病院 (Hospital)’; or ‘東日本大震災 (GEJED)’ [AND] ‘医療 (Healthcare)’. Amazon.co.jp was searched for the books published in Japan from March 2011 to September 2012. The search term used were: ‘東日本大震災 (GEJED)’ [AND] ‘病院 (Hospital)’; or ’東日本大震災 (GEJED)’ [AND] ‘医療 (Healthcare)’. Books that write about hospitals, healthcare, and hospital staff in the time of the GEJED were obtained.

****Eligibility criteria****

**1) Inclusion criteria**

Articles were included in the review if they were: (i) written either in English or in Japanese; (ii) published from March 2011 to September 2012, and (iii) describing the experiences and interviews of rescue teams, on-site health needs, or the prevalence or characteristics of specific diseases, at the time of the GEJED.

**2) Exclusion criteria**

Articles and papers were excluded if they were: (i) the bulletins from universities or private organisations; (ii) abstracts for conferences or lectures; (iii) about clinical interventions or basic science; and (iv) only on the radiation exposure or explosion of nuclear power plant.

## Results

**Search strategy for literature review.**
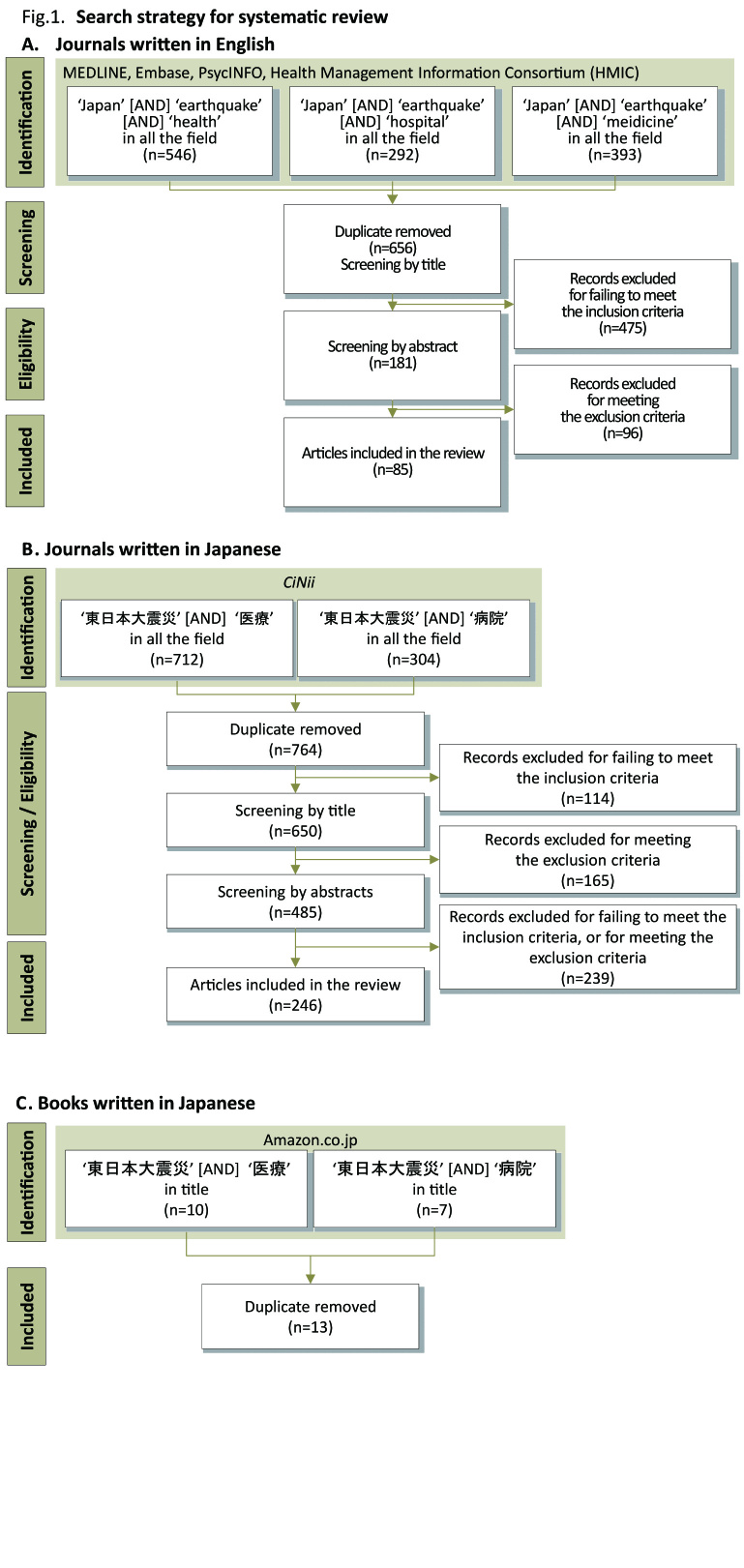
A: Journals written in English B: Journals written in Japanese C: Books written in Japanese

Figure 1 describes the search strategy. The literature search was conducted in September 2012. 85 articles in English, and 246 articles and 13 books in Japanese, were eligible for inclusion in the review (Appendix 1).

The 85 English and the Japanese journal articles were categorised according to types and topics, with some articles addressing more than one. With respect to article types (Fig.2A), for the journals in Japanese, the majority were about the experiences in providing relief activities. The epidemiological research and case reports represent about a third of the articles in English but a lower proportion of the articles in Japanese. As for topics (Fig.2B), many of the English literature wrote on psychiatric care and specific diseases, while disaster medicine was the major topic in Japanese articles.

**Breakdown of the articles (some articles addressing more than one).**
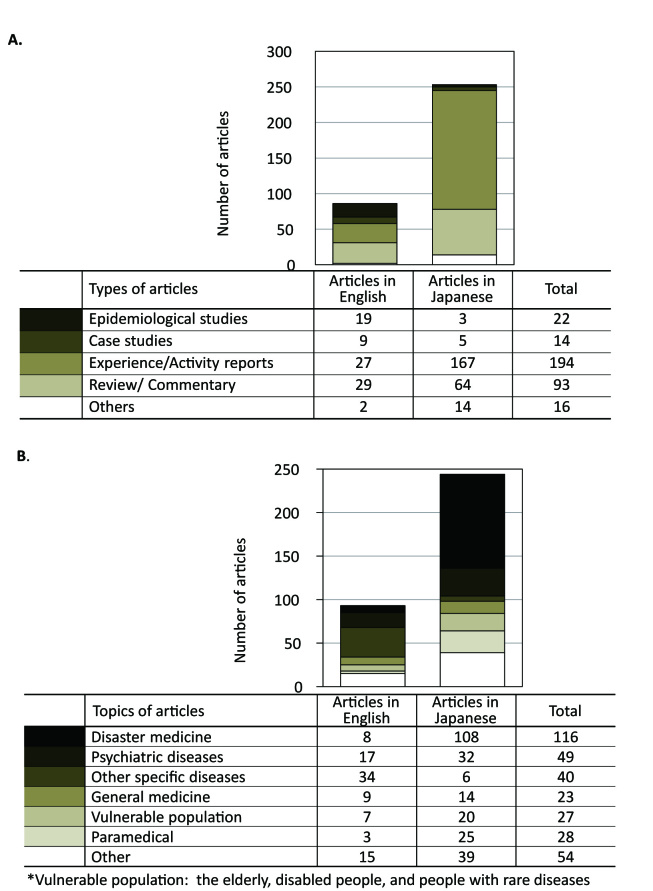
A: Types B: Topics

**Patients’ needs**

Patients’ needs were assessed by the chronology of health impacts, chronic disease, vulnerability and other issues.

**Chronology**

The chronology of impacts are described by the documented effects within 24 hours, 2-10 days, 10-30 days, and more than 30 days. Table 1 summarises the changes in the patients’ needs over time.

**Table 1. Chronology of the major health impacts.** 

**Days from the disaster** ****	**Main healthcare issue**
******** **Within 24 hours**	Few injuries^4^
**2-10days**	Hypothermia^4^^,^ 5 Endogenous diseases^4^ Burning^4^ ‘Tsunami-lung’^5^ Psychiatric shock^4^ Cardio-pulmonary arrest (CPA)^6^ Coronary syndrome^6^ Cerebro-vascular diseases^7^ ‘drug-refugees’^25^
**10-30days**	Respiratory diseases^8^^,^ 9 ^,^ 11 Gastritis^8^^,^ 9 Pressure ulcers^39^ Exacerbation of chronic conditions^24^ Allergic reactions to tsunami debris^8^
**>30days**	Children with allergy^13^ Musculoskeletal disease^2^^,^ 17 ^,^ 38 Deep venous thrombosis (DVT) and pulmonary embolism^15^^,^ 16
**Throughout**	Non-communicable diseases^24^ (hypertension, diabetes, chronic renal failure, cancer, etc.) Pregnancy hypertension^4^^,^ 8 Oxygen-dependent management^11^^,^ 42 Insomnia^6^ Skin-related disorders^18^
**Suspected**	Mental health (depression, posttraumatic stress disorder (PTSD) , and cognitive disorder among the elderly)^41^

<24 hours 

The number of patients seeking healthcare was relatively small on the actual day of the disaster. This can be explained by the fact that the majority of the victims were killed immediately after the earthquake with the injury to death ratio for the disaster being was remarkably low (0.372).^4^

 2-10 days 

The day after the disaster, the number of hospital admissions surged, though injuries remained fewer than anticipated. For example in the Ishinomaki Red-Cross Hospital, among the patients seen within 48 hours of the disaster, injury and crush syndrome represents only 22% of patients.^5^ Instead, the medical teams were preoccupied with saving patients with chronic diseases. This hospital and the Iwate Medical University hospital reported that hypothermia and tsunami-lung (allergic reaction and pneumonia by immersion) were the main severe diseases that were treated.^5^^,^ 6

In the Tohoku University hospital, the incidence of acute coronary syndrome and cardio-pulmonary arrest (CPA) sharply increased.^7^ Freezing temperature, stresses from evacuation and frequent aftershocks may have over-activated patients’ sympathetic nervous system, leading to hyper viscosity and hypertension. Additionally, the Ishinomaki Red-Cross hospital determined that the incidence rate of cerebro-vascular diseases also increased compared to the previous year.^8^

10-30 days

At this time it was found that respiratory tract diseases spread both in hospitals^9^ and an evacuation centre in Miyako City.^10^ The sludge from the tsunami was found to be highly contaminated with chemical and bacteria^11^ which may have contributed to a large number of shelter-acquired pneumonia (SAP)^12^ and allergic reactions.^8^ In Ishinomaki City, the patients with chronic pulmonary failure were especially vulnerable to these infections.^13^

 30days-

A month after the earthquake, deterioration of allergic conditions among children was reported by Miura et al.^14^ Poor hygienic status due to a very limited water supply,^15^ prolonged mental stress, and exposure to the allergen such as mites, the sludge from the tsunamis, and household pets at the evacuation centres, were the main cause of the deterioration.^14^ Among the adults, deep venous thrombosis (DVT) resulting from long-term immobility and dehydration was a concern at the evacuation centres. On-site screening rounds by university hospitals revealed high prevalence of DVT (10-25%)^15, 16^ , especially in those who had received leg injuries.^16^ Living on the floor at the evacuation centres also worsened the trend.^15^ Chronic subdural hematoma (CSH) and unstable pelvic fractures after immersion were also reported by Numagami et al^8^ and Ishii.^17^

Throughout the disaster period

An increase in non-fatal conditions was reported in some articles. For example, 15% of the patients who visited temporary clinics had skin problems.^19^ Others noted an increase in muscle pain, constipation,^18^ insomnia, headache,^7^ and vision problems.^20^ As these patients often refrained from seeing doctors due to the lack of transportation infrastructure, the numbers might be underestimated.^20^

 Success in infection prevention

The poor hygienic status due to lack of water for hand-washing and the crowded living conditions raised concerns about outbreaks of highly contagious infections. Although sporadic cases of gastroenteritis, a case of measles in an otherwise healthy foreigner,^8^ several cases of tetanus,^7^ and increase in the latent tuberculosis due to the dysfunction of negative pressure rooms^21^ were reported, only one outbreak of influenza^21^ had been reported up to September 2012. This might be attributed to the vigorous public health efforts, including the Daily Surveillance for Outbreak Detection,^23^^,^ 24 which was a collaborative activity in a university hospital with infectious disease clinical consultation. This included infection control educational programmes and training and infection control interventions. When these were reported to the local government regions, it assisted in defining actions required against infectious diseases that were identified.

**Non-communicable diseases**

Most of the patients who visited temporary clinics presented with non-communicable diseases.^25^ One reason was the aging profile of the population in the disaster area, many of whom had pre-existing diseases. Another reason was a huge number of ‘drug refugees’,^26^ people who had their medication and prescriptions washed away. Adding to this, living in the evacuation centres caused exacerbation of existing illnesses.

 Hypertension

Hypertension including pregnancy hypertension^9^ was a significant problem within a few days of the disaster. In addition to the pre-existing disease, it is thought that activation of the sympathetic nervous system by frustration and disruption of circadian rhythm through poor sleep quality^27^ may have led to the poor blood pressure control.

 Diabetes

Deterioration of glycaemic control in diabetes after the disaster was reported by Ogawa et al,^28^ in part due to loss of medication, even though the average body mass index (BMI) decreased among non-insulin dependent diabetics. The deterioration was more significant when patients were affected by the tsunami, suggesting that psychological effects and loss of prescription were part of the reason for this poor outcome.

 Problems in the supply of foods

For patients with hypertension, diabetes, and chronic renal failure usually on special diets, the enforced diet in the evacuation shelters which was high in sodium and potassium worsened their conditions.^29^ For the diabetic patients, taking drugs that may cause hypoglycaemia put patients at increased risk in situations where meals were supplied at infrequent intervals. Children with food allergies also suffered with the lack of low-allergic foods.^14^

 Problems with living environments

As Japanese people are used to sleeping on the floor, most of the evacuation centres were not equipped with beds. However, in crowded environment without heating systems, living on the floor increases the risks of hypothermia and muscle stiffness. These conditions may have contributed to the high prevalence of disuse syndrome, muscle weakness as a result of inactivity, which affected 30% of the evacuees.^3^

Psychological reactions

A huge disaster poses a concern about psychiatric disorders such as depression, post-traumatic stress disorder (PTSD), and cognitive disorder among the elderly. However, no articles so far report the actual incident rate of these illnesses in the disaster area, though an increase in the complaints of anxiety, irritation, and fatigue^30^ and a worsening in a psychological distress score^31^ were reported.

**** **Vulnerable people**

In a disaster, ‘a harvesting effect’, that is, a selective mortality among the frailest individuals,^32^ often occurs. The government had set up guidelines on the provision of designated shelters for those in need of care.^33^ However, in the GEJED, the number of the vulnerable far exceeded the capacity of these shelters. As a result, the care of these people became a serious issue in the disaster area.

 The elderly

In the GEJED, many of the victims were those requiring health and social care. Among the healthcare facilities destroyed by the tsunamis, 40% were special elderly care nursing homes presumably because they had been located at areas at higher risk.^34^ Yoshioka^35^ reported that 316 people in social welfare facilities died with 178 people missing, two-thirds of whom were above 60. Other disabled elderly living alone were found deceased at home.^36^ Even for those who reached evacuation shelters, living on the floor without heating systems^37^ seemed very difficult. For instance 85% of the tsunami-related pneumonia patients who needed hospitalisation were over 70, 45% of whom were from evacuation centres.^38^ Many of these patients had both prolonged swallowing reflex and low sensitivity to cough reflex,^9^^,^ 11 which are the risk factors of aspiration pneumonia. Secondary immobility also became a problem. Around 60% of the elderly in the shelters were reported to be suffering from disuse syndrome a month after the disaster.^39^ In the week following the earthquake, 7.7% of patients in one hospital developed severe pressure ulcers.^40^ Dementia, depression, and disquiet were also observed and the authors stated that this may have been due to an inability to adapt to changes in lifestyle.^39^

 The mentally ill

Many hospitals with psychiatric care beds were severely affected: 3 hospitals collapsed and were destroyed and 5 were closed.^41^ The National Center of Neurology and Psychiatry (NCNP) promptly transported the inpatients out of the earthquake/tsunami damaged region. They also supplied drugs, provided information, and dispatched mental health care teams to the disaster struck area.^41^ However, many outpatients were left untreated because of loss of medical records, loss of patients’ family members who had cared for them, and the disruption of transportation.^42^ In some places, stigma attached to mental illness remained deeply rooted, which prevented patients in evacuation centres from seeking help.^42^

 Those with disabilities

Others who were often neglected were those with disabilities, who were found to be two times more at risk of losing their lives compared with healthy people.^43^ Those who needed home oxygen therapy^12^ and respirators^43^ suffered from the long-term disruption of power supply. In some areas, a registration system for those who need relief in times of disaster had been established, but some of the staff in charge did not make contact with those who had registered, for unknown reason.^44^

 Others

There were concerns about pregnant women, patients with cancer, and even overseas nationals living in the earthquake/tsunami region,^39^ but no reports so far have been made on the needs amongst these groups.

**Successes and future improvement in rescue activities** ****

The Disaster Medical Assistance Teams (DMATs), aimed at deploying medical rescue teams to a disaster area within 48 hours, were established in 2005, based on the lessons from the Great Hanshin-Awaji Earthquake (1995).^3^ Their response was prompt, and 15,000 professionals in total were dispatched to Tohoku area within 2 days of the quake.^45^ Other Japanese medical teams were also dispatched as rescue teams in the early stage of the disaster.

Even so, coordination between the local healthcare staff and the rescue teams was often reported to be poor.^37^ Lack of the communication tools due to power outages caused confusion in handovers between these teams, reducing the appropriate mobilisation of resources.^38^ Another problem was that most of the teams aimed at providing emergency care only for the first three days, even though there was a significant need for primary care and care for chronic conditions.^24^ The medical rescue teams tasks were often limited: for example, they were not allowed to prescribe.^46^ Responding to this situation, the Japan Primary Care Association dispatched in total 678 staff to provide primary care without task limitations from March to September in 2011.^47^ So far this response has not had a published evaluation, but it is thought that the numbers of staff to support primary care may not have been sufficient.

## Discussion

This is the first literature review of the health needs at the time of GEJED. To collect richer information on the needs among the vulnerable population, the ‘grey’ literature written in the local language (Japanese) were useful. However, few hard facts and figures were available in either language.

In addition, there were relatively small numbers of published articles focusing on building evidence-base knowledge on the impact of the GEJED. Both researchers and public health authorities should consider taking up the challenge of conducting retrospective research as well as prospective surveillance to obtain a more complete picture of the GEJED in order to prepare for future mega-disasters in Japan or other countries worldwide.

The greatest success in this disaster was the prompt reactions of the medical rescue teams. Even so, belated recognition of the health needs and health impacts were often reported. Above all, the most vulnerable were the least risk-assessed. As can be seen in Figure 2, only 27 out of 331 articles addressed issues about the vulnerable population including geriatric care and care for the disabled. This lack of recognition may have led to the underestimation of the size of these populations in contingency plans prior to the GEJED. Considering the increasing number of elderly and mentally ill, these people should be identified as a major target for relief activities. Therefore, to ‘develop systems of indicators of disaster risk and vulnerability…that will enable decision-makers to assess the impact of disasters’, as is stated in the *Hyogo Framework of Action* 2005-2015: Building the Resilience of nations and Communities,^47^ is strongly recommended.**

The complexity of crisis management in this disaster posed the need for an assessment of health system capacity and public health emergency preparedness.^48^ It is strongly recommended that future plans should put more focus on collecting data both from within and outside of the health sector during, and in the recovery from, a disaster. A comprehensive guideline for this system-wide approach, such as the toolkit for assessing health-systems capacity for crisis management by WHO Regional Office for Europe^50^^,^ 51 will be useful to share the knowledge with global and multi-disciplinary teams.

The proposed recommendations based on this literature review are listed in Box 1.

**Box 1. Recommendations for future preparedness.** 

Recommendations
1. Public health research after disasters frequently lack baseline data from before the event. Disease risk reduction for health needs requires baseline data and health system to provide information as for disaster preparedness, maintaining, analysis and eventual evaluation of the health impacts.
2. Health needs of the vulnerable including the elderly and those with chronic conditions can predominate the health care needs after disaster. To enhance capacities for the most vulnerable populations, health impact data at the time of the disaster should be analysed with regard to the socio demographic background of the patients.
3. Lack of shared information systems could have reduced cooperation between relief teams. For effective and timely disaster response, a robust data collection and dissemination system should be established so that the resources are appropriately allocated, even at times of power outages and water supply failures.
4. Few scientific peer reviewed papers on health needs at the time of the disaster have been identified in this review. Public health researchers and authorities should be encouraged to take the initiative to fill gaps between academic knowledge and onsite needs at the time of disasters.

**Limitations**

The articles in this literature review came from only a short time period of March 2011 to September 2012. Little objective data, such as epidemiological surveillance or studies, was found making the recognition of the full health impact difficult to assess. In addition, this review might have failed to establishneeds of the so far undocumented minorities, such as the overseas nationals and those with rare diseases. It is recommended that in-depth surveillance and quantitative analysis are conducted to overcome these limitations.

**Conclusion**

This paper is the first literature review on the health needs and relief activities following the GEJED. It has demonstrated the huge array of peer review and grey literature already published on the health impacts of the GEJED in the Japanese and English literature. The review has identified a chronology of patients’ health needs from the immediate (i.e. within 24 hours) needs to those identified up to 30 days or longer. The range of needs identified included relatively little treeatment for immediate crush injury or trauma, the tsunami-lung health impacts, SAP issues, DVT risks and the significant impact on non-communicable diseases including hypertension, diabetes, renal disease and mental health. However, concern about the need for a public health leading health systems approach has been identified because of the complexity of crisis preparedness.

Accumulation of experience, evaluation of the rescue activities, and the establishment of new contingency plans that fit the health needs, will be the key to more efficient and effective relief activities in the future.

## Competing interest

The authors have declared that no competing interests exist.

## Appendix 1

### Appen**dix 1. List of the referred literature**

**I. Journal articles written in English**

(1) Japan Earthquake Linked to Blood Pressure Rise in CKD. Nephrology Times 2012 September;5(9):3.

(2) Mental health care and East Japan Great Earthquake. Psychiatry & Clinical Neurosciences 2011 April;65(3):207-212.

(3) Ahearn A. Chemical contamination in Tohoku, with Lizzie Grossman and Winnie Bird. Environ Health Perspect 2011;119(7).

(4) Akabayashi A, Kodama S. Lessons from Japan's March 2011 Earthquake Regarding Dialysis Patients. Therapeutic Apheresis & Dialysis 2011 June;15(3):334.

(5) Akiba S. Our response in the aftermath of the great Tohoku-Kanto Earthquake and Tsunami. Journal of Epidemiology 2011 2011;21(4):237-239.

(6) Asayama K, Staessen JA, Hayashi K, Hosaka M, Tatsuta N, Kurokawa N, et al. Mother-off spring aggregation in home versus conventional blood pressure in the Tohoku Study of Child Development (TSCD). Acta Cardiol 2012 2012;67(4):449-456.

(7) Bird WA, Grossman E. Chemical aftermath: Contamination and cleanup following the tohoku earthquake and tsunami. Environ Health Perspect 2011 July 2011;119(7):A290-A301.

(8) Blair, Gavin. Japan's suicide rate is expected to rise after triple disasters in March. BMJ 2011 September 17;343:5839.

(9) Coleman CN, Simon SL, Noska MA, Telfer JL, Bowman T. Disaster preparation: Lessons from Japan. Science 2011 17 Jun 2011;332(6036):1379.

(10) Coleman CN, Whitcomb J, R.C, Miller CW, Noska MA. Commentary on the combined disaster in Japan. Radiat Res 2012 January 2012;177(1):15-17.

(11) Ebisawa K, Yamada N, Okada S, Suzuki Y, Satoh A, Kobayashi M, et al. Combined legionella and escherichia coli lung infection after a tsunami disaster. Internal Medicine 2011 2011;50(19):2233-2236.

(12) Furukawa K, Ootsuki M, Kodama M, Arai H. Exacerbation of dementia after the earthquake and tsunami in Japan. J Neurol 2012 June;259(6):1243-1243.

(13) Fuse A, Igarashi Y, Tanaka T, Kim S, Tsujii A, Kawai M, et al. Onsite medical rounds and fact-finding activities conducted by Nippon Medical School in Miyagi prefecture after the Great East Japan Earthquake 2011. Journal of Nippon Medical School 2011 2011;78(6):401-404.

(14) Fuse A, Shuto Y, Ando F, Shibata M, Watanabe A, Onda H, et al. Medical relief activities conducted by Nippon Medical School in the acute phase of the Great East Japan Earthquake 2011. Journal of Nippon Medical School 2011 2011;78(6):397-400.

(15) Hamasaki T, Chishiro T. Medical support and acute stress disorder in medical staff after providing medical support for people affected by the great east Japan earthquake. Acad Emerg Med 2012 June 2012;19(6):714.

(16) Hatta M, Endo S, Tokuda K, Kunishima H, Arai K, Yano H, et al. Post-Tsunami Outbreaks of Influenza in Evacuation Centers in Miyagi Prefecture, Japan. Clinical Infectious Diseases 2012 January 01;54(1):e5-e7.

(17) Hayashi K, Tomita N. Lessons learned from the great East Japan earthquake: impact on child and adolescent health. Asia-Pacific Journal of Public Health 2012 Jul;24(4):681-688.

(18) Igusa R, Narumi S, Murakami K, Kitawaki Y, Tamii T, Kato M, et al. Escherichia coli pneumonia in combination with fungal sinusitis and meningitis in a tsunami survivor after the Great East Japan earthquake. Tohoku J Exp Med 2012;227(3):179-184.

(19) Iijima K, Shimokado K, Takahashi T, Morimoto S, Ouchi Y, Members of JGS Disaster Supportive Center. Actions of the Japan Geriatric Society in response to the 2011 off the Pacific Coast of Tohoku Earthquake: First report. Geriatrics & Gerontology International 2011 October;11(4):525-526.

(20) Inoue Y, Shozushima T, Koeda Y, Nakadate T, Fujino Y, Onodera M, et al. Tsunami lung. Journal of Anesthesia 2012 April 2012;26(2):246-249.

(21) Ishii TMD, Harasawa KMD, Tanimoto TMD. Drowning. N Engl J Med 2012 August 23;367(8):777.

(22) Kanamori H, Aso N, Weber DJ, Koide M, Sasaki Y, Tokuda K, et al. Latent Tuberculosis Infection in Nurses Exposed to Tuberculous Patients Cared for in Rooms without Negative Pressure after the 2011 Great East Japan Earthquake. Infection Control & Hospital Epidemiology 2012 February;33(2):204-206.

(23) Kanamori H, Kunishima H, Tokuda K, Kaku M. Infection Control Campaign at Evacuation Centers in Miyagi Prefecture after the Great East Japan Earthquake. Infection Control & Hospital Epidemiology 2011 August;32(8):824-826.

(24) Kario K. Disaster hypertension-Its characteristics, mechanism, and management. Circulation Journal 2012 2012;76(3):553-562.

(25) Kato Y, Uchida H, Mimura M. Mental health and psychosocial support after the Great East Japan Earthquake. Keio J Med 2012 March 2012;61(1):15-22.

(26) Kawakami Y, Tagami T, Kusakabe T, Kido N, Kawaguchi T, Omura M, et al. Disseminated aspergillosis associated with tsunami lung. Respir Care 2012 Oct;57(10):1674-1678.

(27) Kazama JJ, Narita I. Earthquake in Japan. The Lancet 2011 May 14-20, 2011;377(9778):1652-1653.

(28) Keim ME. The public health impact of tsunami disasters. American journal of disaster medicine 2011 2011;6(6):341-349.

(29) Kim Y. Great East Japan earthquake and early mental-health-care response. Psychiatry & Clinical Neurosciences 2011 Oct;65(6):539-548.

(30) Kimura M, Yamamoto R, Oku S. Interim report of healthcare delivery after east Japan earthquake-tsunami disaster--does EHR help?. Methods Inf Med 2011;50(5):393-396.

(31) Kobayashi S, Hanagama M, Yamanda S, Yanai M. Home oxygen therapy during natural disasters: Lessons from the great East Japan earthquake. European Respiratory Journal 2012 01 Apr 2012;39(4):1047-1048.

(32) Kohsaka S, Endo YBS, Ueda I, Namiki J, Fukuda K. Necessity for Primary Care Immediately After the March 11 Tsunami and Earthquake in Japan. Arch Intern Med 2012 February 13;172(3):290-291.

(33) Kotozaki Y, Kawashima R. Effects of the Higashi-Nihon Earthquake: Posttraumatic stress, psychological changes, and cortisol levels of survivors. PLoS ONE 2012 Apr;7(4):Art e34612.

(34) Kyutoku Y, Tada R, Umeyama T, Harada K, Kikuchi S, Watanabe E, et al. Cognitive and psychological reactions of the general population three months after the 2011 tohoku earthquake and tsunami. PLoS ONE 2012 Article Number: e3;7(2):ate of Pubaton: 08 Feb 2012.

(35) Lim GB. PUBLIC HEALTH: Cardiovascular diseases after the Great East Japan Earthquake. Nature Reviews Cardiology .

(36) Liu M, Kohzuki M, Hamamura A, Ishikawa M, Saitoh M, Kurihara M, et al. How did rehabilitation professionals act when faced with the Great East Japan earthquake and disaster? Descriptive epidemiology of disability and an interim report of the relief activities of the ten Rehabilitation-Related Organizations. J Rehabil Med 2012 May;44(5):421-428.

(37) Matsumoto M, Inoue K. Earthquake, tsunami, radiation leak, and crisis in rural health in Japan. Rural and remote health 2011 2011;11:1759.

(38) Matsusaka K, Yamaya M, Oikawa T. Management of bedridden patients during an earthquake in Japan. Gerontology 2012 December 2011;58(1):60-61.

(39) Meguro K. International Report: Local response following the Great East Japan Earthquake 2011. Neurology 2011 July 19;77(3):e12-e15.

(40) Merin O, Blumberg N, Raveh D, Bar A, Nishizawa M, Cohen-Marom O. Global responsibility in mass casualty events: the Israeli experience in Japan. American journal of disaster medicine 2012 2012;7(1):61-64.

(41) Morimoto S, Iijima K, Kuzuya M, Hattori H, Yokono K, Takahashi T. Guidelines for Non-Medical Care Providers to Detect Illnesses in Elderly Evacuees After the 2011 Earthquake Off the Pacific Coast of Tohoku. J Am Geriatr Soc 2011 November;59(11):2189-2191.

(42) Moszynski, Peter. Death toll climbs and healthcare needs escalate in Japan. BMJ 2011 March 26;342:1859.

(43) Murata S, Hashiguchi N, Shimizu M, Endo A, Omura N, Morita E. Skin disorders and the role of dermatologists after the tsunami in Japan. Journal of the European Academy of Dermatology & Venereology 2012 July;26(7):923-924.

(44) Nagamatsu S, Maekawa T, Ujike Y, Hashimoto S, Fuke N. The earthquake and tsunami - observations by Japanese physicians since the 11 March catastrophe. Critical Care 2011;15(3).

(45) Nakano M, Kondo M, Wakayama Y, Kawana A, Hasebe Y, Shafee MA, et al. Increased incidence of tachyarrhythmias and heart failure hospitalization in patients with implanted cardiac devices after the great East Japan Earthquake disaster. Circulation Journal 2012 2012;76(5):1283-1285.

(46) Nangaku M, Akizawa T. Diary of a Japanese nephrologist during the present disaster. Kidney Int 2011 May 2011;79(10):1037-1039.

(47) Nishi D, Koido Y, Nakaya N, Sone T, Noguchi H, Hamazaki K, et al. Peritraumatic distress, watching television, and posttraumatic stress symptoms among rescue workers after the Great East Japan Earthquake. PLoS ONE 2012 Apr;7(4):Art e35248.

(48) Nishizawa M, Hoshide S, Shimpo M, Kario K. Disaster hypertension: experience from the great East Japan earthquake of 2011. Curr Hypertens Rep 2012 Oct;14(5):375-381.

(49) Noda, Fumitaka. A current report on the impact of the recent earthquake, tsunami and nuclear hazard in northeastern Japan. Asia-Pacific Psychiatry 2011 June;3(2):96-97.

(50) Oda M, Watanabe H, Oda E, Tomita M, Obata H, Ozawa T, et al. Rise in international normalized ratio after a catastrophic earthquake in patients treated with warfarin. Int J Cardiol 2011 06 Oct 2011;152(1):109-110.

(51) Ogawa S, Ishiki M, Nako K, Okamura M, Senda M, Sakamoto T, et al. Effects of the Great East Japan Earthquake and huge tsunami on glycaemic control and blood pressure in patients with diabetes mellitus. BMJ Open 2012;2(2).

(52) Ooe Y, JMAT Osaka Team N, 25. Report on support activity for the East Japan Great Earthquake (May 27-29, 2011). Japan-Hospitals 2012 Jul(31):63-69.

(53) Oshima CR, Yuki K, Uchida A, Dogru M, Koto T, Ozawa Y, et al. The Vision van, a mobile eye clinic, Aids relief Efforts in Tsunami-Stricken areas. Keio J Med 2012 March 2012;61(1):10-14.

(54) Ota H, Kawai H. An unusual case of pleural empyema in a tsunami survivor. Asian Cardiovascular & Thoracic Annals 2012 Jun;20(3):344-346.

(55) Otani Y, Ando T, Atobe K, Haiden A, Kao S-, Saito K, et al. Comparison of two large earthquakes: The 2008 Sichuan earthquake and the 2011 east Japan Earthquake -from a student discussion session in Beijing, 2011. Keio J Med 2012 March 2012;61(1):35-39.

(56) Procter NG, Crowley TMNS,. A Mental Health Trauma Response to the Japanese Earthquake and Tsunami. Holist Nurs Pract 2011 May/June;25(3):162-164.

(57) Sakai T. What have we learned from Japan's major earthquake, tsunami and nuclear incident?. World hospitals and health services : the official journal of the International Hospital Federation 2011 2011;47(4):10-12.

(58) Sato T, Ichioka S. Pressure ulcer occurrence following the great East Japan Earthquake: observations from a disaster medical assistance team. Ostomy Wound 2012 Apr 2012;58(4):70-75.

(59) Satoh M, Kikuya M, Ohkubo T, Imai Y. Acute and Subacute Effects of the Great East Japan Earthquake on Home Blood Pressure Values. Hypertension 2011 December;58(6):e193-e194.

(60) Satomi S. The Great East Japan Earthquake: Tohoku University Hospital's efforts and lessons learned. Surg Today 2011 September 2011;41(9):1171-1181.

(61) Stone K. NerveCenter: Japan's neurological community contends with triple disaster. Ann Neurol 2011 June 2011;69(6):A14-A15.

(62) Sugimoto A, Krull S, Nomura S, Morita T, Tsubokura M. The voice of the most vulnerable: Lessons from the nuclear crisis in Fukushima, Japan. Bull World Health Organ 2012 August 2012;90(8):629-630.

(63) Suzuki M, Uwano C, Ohrui T, Ebihara T, Yamasaki M, Asamura T, et al. Shelter-Acquired Pneumonia After a Catastrophic Earthquake in Japan. J Am Geriatr Soc 2011 October;59(10):1968-1970.

(64) Suzuki Y, Kim Y. The Great East Japan earthquake in 2011; toward sustainable mental health care system. Epidemiology and Psychiatric Sciences 2012 Mar;21(1):7-11.

(65) Suzuki Y, Weissbecker I. Post-disaster mental health care in Japan. Lancet 2011 23 Jul 2011;378(9788):317.

(66) Takahashi T, Goto M, Yoshida H, Sumino H, Matsui H. Infectious Diseases after the 2011 Great East Japan Earthquake. Journal of Experimental and Clinical Medicine 2012 February 2012;4(1):20-23.

(67) Takayama S, Kamiya T, Watanabe M, Hirano A, Matsuda A, Monma Y, et al. Report on disaster medical operations with acupuncture/ massage therapy after the great ast japan arthquake. Integrative Medicine Insights 2012 2012;2012(7):1-5.

(68) Takeda M. Mental health care and East Japan great earthquake. Psychiatry Clin Neurosci 2011 Apr;65(3):207-212.

(69) TAKEDA M, TANAKA T. The Great Eastern Japan Earthquake and psychogeriatric services. Psychogeriatrics 2011 December;11(4):191-195.

(70) Tanaka Y, Yamato A, Yaji N, Yamato M. A simple stock of optical glasses for a catastrophic disaster: Eyewear donations after the 2011 pacific coast Tohoku earthquake. Tohoku J Exp Med 2012;227(2):93-95.

(71) Tayama J, Ichikawa T, Eguchi K, Yamamoto T, Shirabe S. Tsunami damage and its impact on mental health. Psychosomatics: Journal of Consultation Liaison Psychiatry 2012 Mar-Apr;53(2):196-197.

(72) Tokudome S, Nishi N, Tanaka H. Towards a better National Health and Nutrition Survey in Japan. The Lancet 2012 March 2012;379(9821):e44.

(73) Tsubokura M, Horie S, Komatsu H, Tokiwa M, Kami M. The impact of the Great Tohoku Earthquake on the dialysis practice in the disaster-stricken area. Hemodialysis International 2012 April;16(2):320-321.

(74) Tsuiki S, Shiga T, Maeda K, Matsuzaki-Stromberger R, Inoue Y. A dentist's role: prevention of snoring at temporary refuges for victims of the East Japan earthquake and the Fukushima Daiichi Nuclear Power Plant accident on March 11, 2011. Sleep & Breathing 2012 Sep;16(3):587-589.

(75) Tsuji S, Mateen FJ. Earthquakes and neurological care. European Journal of Neurology 2012 February;19(2):185-186.

(76) Ueda S, Hanzawa K, Shibata M, Suzuki S. High prevalence of deep vein thrombosis in tsunami-flooded shelters established after the Great East-Japan earthquake. Tohoku J Exp Med 2012;227(3):199-202.

(77) Valeo T. An Earthquake, A Tsunami, A Nuclear Disaster: Japan's Neurologists on the Front Lines. Neurology Today 2011 April 21;11(8):1,11,14-15.

(78) Wiwanitkit V. Post-crisis: earthquake, tsunami, radiation leak and rural health crisis in Japan. Rural and remote health 2011 2011;11:1770.

(79) Yamamoto A. Experiences of the Great East Japan Earthquake March 2011. Int Nurs Rev 2011 September;58(3):332-334.

(80) Yamamoto T, Kato M, Shirabe S. Life, health, and community in a tsunami-affected town. The Lancet 2011 July 23-29, 2011;378(9788):318.

(81) Yamazaki M, Minami Y, Sasaki H, Sumi M. The psychosocial response to the 2011 Tohoku earthquake. Bull World Health Organ 2011 September 2011;89(9):623.

(82) Yambe T, Shibata M, Sumiyoshi T, Mibiki Y, Osawa N, Katahira Y, et al. Medical Responses Following the Sendai Quake (East Japan Earthquake, March 11, 2011). Artif Organs 2012 August;36(8):760-763.

(83) Yanagimoto S, Haida M, Suko M. An asthma patient with steroid-resistant decrease in peak expiratory flow after the great east Japan earthquake showing spontaneous recovery after 1 month. Internal Medicine 2012 2012;51(12):1631-1634.

(84) Yasunari TRNM, M.N.Sc, Nozawa MRNM, Nishio RRNM, M.N.Sc, Yamamoto ARNM, Takami YRNM, M.N.Sc. Development and evaluation of 'disaster preparedness' educational programme for pregnant women. Int Nurs Rev 2011 September;58(3):335-340.

(85) Yatabe J, Yatabe MS, Taguchi FRD, Izumi IRD, Sato ALPN, Hoshi MLPN, et al. Outcomes of Emergency Reduction of Tube Feeding in Hospitalized Elderly Adults During the Aftermath of the Great East Japan Earthquake. J Am Geriatr Soc 2012 April;60(4):804-805.

** II. Journals articles written in Japanese**

(1) 東日本大震災看護・介護支援. 看護と介護 : 北海道勤労者医療協会看護雑誌 2012 04;38:10.

(2) 特集東日本大震災救急患者が45%増 : 受診我慢し重症化 : 被災沿岸5病院. 厚生福祉 2012 03/13(5888):2.

(3) 東日本大震災その後石巻赤十字病院. 病院 2012 03;71(3):169.

(4) 栞田但. Revival of Public Hospitals in Iwate Prefecture after the Great East Japan Earthquake : Lessons for Improving Sustainability of Medical Delivery in Local Agricultural Districts. 医療と社会 2012 07;22(2):157.

(5) Fukudo S, Shoji T, Endo Y, Kano M, Tamura D, Morishita J, et al. Stress at the Tohoku Earthquake and Tsunami : Report from Sendai-Miyagi(Assistance Programs of the Great East Japan Earthquake). Japanese Journal of Psychosomatic Medicine 2012 05/01;52(5):388.

(6) Hashizume M, Suzuki Y, Tsuboi K. Mental Health Care in Crisis and Disaster(Assistance Programs of the Great East Japan Earthquake). Japanese Journal of Psychosomatic Medicine 2012 05/01;52(5):365.

(7) Hirata A, Nishi K, Sekine C. Television Stays as a Deep-Rooted Information Tool While Portal Sites Increase Their Presence : From the Public Opinion Survey on Information and Media Use. The NHK monthly report on broadcast research 2012 07;62(7):44.

(8) Iseki K, Hayashida A, Seino K, Iwashita Y, Shinozaki K. Clinical services in the emergency department of Yamagata University Hospital after the Great East Japan Earthquake. Bulletin of the Yamagata University.Medical science 2012 02/25;30(1):1.

(9) Minoura T, Yanagida N, Watanabe Y, Yamaoka A, Miura K. THE EFFECTS OF GREAT EAST JAPAN EARTHQUAKE ON PATIENTS WITH FOOD ALLERGY IN MIYAGI PREFECTURE. Japanese Journal of Allergology 2012 05/30;61(5):642.

(10) Murakami N. Holistic Care for Loss and Grief Resulting from Disasters(Assistance Programs of the Great East Japan Earthquake). Japanese Journal of Psychosomatic Medicine 2012 05/01;52(5):373.

(11) NAKAMURA H. JAOT's support activities for survivors of the Great East Japan Earthquake. 作業療法 = The Journal of Japanese Occupational Therapy Association 2011 08/15;30(4):394.

(12) Noda K. THE EXPERIENCE OF COOPERATING WITH JAPANESE NURSE PRACTITIONER AND PHYSICIAN ASSISTANT IN THE DISASTER-HIT AREAS. Journal of Japan Surgical Society 2011 07/01;112(4):288.

(13) Numagami Y, Kikuchi T, Ishikawa S, Aizawa M, Hino M, Ishibashi S, et al. Neurosurgical Service during the Great East Japan Earthquake Disaster : Events at Ishinomaki Red Cross Hospital, a Reference Hospital in the Affected Area. Japanese journal of neurosurgery 2011 12/20;20(12):904.

(14) Rokkaku R. A Coping on Mito Day Service at the Time of the Great East Japan Earthquake(The Great East Japan Earthquake Support Project). journal of Japan Academy of Gerontological Nursing 2011 11/30;16(1):132.

(15) SATO H. The necessity of understanding the actual situation of children and adults who are disabled and require home care with medical treatment: the case of the Tokyo metropolitan area during the Great East Japan earthquake. Core ethics : コア・エシックス 2012;8:183.

(16) Shinozaki K, Suzuki W, Kimura A, Tsuchiya T, Miura S, Hayashida A, et al. A report of the activities by Yamagata University Disaster Medical Assistant Team in the acute term after the East Japan Great Earthquake. Bulletin of the Yamagata University.Medical science 2012 02/25;30(1):33.

(17) SHIOKAWA H. An Experience of Natural Disaster Medical Assistance in Tohoku. Psychiatria et neurologia paediatrica Japonica 2011 06/30;51(2):119.

(18) Takahashi A. WORKING FOR JAPANESE NP/PA AFTER THE GREAT TOHOKU NATURAL DISASTER : TALENT OF NP/PA ON PERSPECTIVE OF NURSE. Journal of Japan Surgical Society 2011 09/01;112(5):354.

(19) TAKAYAMA S, OKITSU R, IWASAKI K, WATANABE M, KAMIYA T, HIRANO A, et al. The Role of Oriental Medicine in the Great East Japan Earthquake Disaster. Kampo medicine 2011 09/20;62(5):621.

(20) TANAKA S. The Great East Japan Earthquake and Support for Children with Severe Motor and Intellectual Disabilities. Japanese journal for the problems of the handicapped 2012 08;40(2):44.

(21) YAMAKATA D. The situation and deliberation of medical ICT network in disaster time and recovery period. IEICE technical report.Social Implications of Technology and Information Ethics 2011 10/11;111(240):13.

(22) YODA T, RAKUE Y. Response to the earthquake, tsunami and nuclear crisis in Japan-Disaster Leadership in Action, from Special Forum at Harvard School of Public Health. The Journal of Tokyo Medical University 2011 07/30;69(3):389.

(23) シュナックジ, 竹中万. 東日本大震災でソーシャルメディアが果たした役割 Twitterから読む災害医療の現場 (東日本大震災から学ぶ) -- (医師が見た災害医療の現場地域医療再生への願い). Medical information express 2011 05;39(6):30.

(24) 上原鳴, 小泉俊. 新着情報『東日本大震災』の現場からいま何が必要か?--災害によるシステムの破壊から被災者をまもるために--上原鳴夫東北大学大学院教授に聞く. The Japanese journal of quality and safety in healthcare 2011;6(2):261.

(25) 中谷祐. 東日本大震災と精神科病院 (特集東日本大震災と精神科病院). Journal of Japanese Association of Psychiatric Hospitals 2011 10;30(10):951.

(26) 丸川征, 森典, 細矢光. 座談会医療サービスの情報化で、激甚災害に備える(第3回)東日本大震災の教訓を生かし、ICTを活用して災害時医療支援をより効率的に行うには. 月刊基金 : 医療保険を支えるネットワークマガジン 2012 02;53(2):6.

(27) 五十嵐豊, 萩原純, 大村真. Geriatric Patient Transportation from Disaster-Affected Hospitals in the Great East Japan Earthquake. 日本集団災害医学会誌 2012 07;17(1):291.

(28) 五十嵐隆. 大震災後の日本小児科学会の小児保健・小児医療への取り組み (特集大災害と母子保健) -- (東日本大震災から学ぶ災害時の母子の保健と福祉). 母子保健情報 2011 11(64):1.

(29) 五十洲剛, 村川雅. Management of the Operating Room at the Time of Emergency Outbreak : The Experience of the 2011 Off the Pacific Coast of Tohoku Earthquake. 麻酔 2012 03;61(3):245.

(30) 井上孝, 山口芳. Let’s start! 災害医療(第36回)東日本大震災から学ぶこと(3)東京DMATの活動. 救急医療ジャーナル : 救急医療専門情報誌 2012 02;20(1):44.

(31) 今田隆. 東日本大震災被害の実態と取り組み--当院宮城厚生協会坂総合病院]の経験を通じて (特集地震、津波…その時医療は--東日本大震災から現在まで). 月刊保団連 2011 10:15.

(32) 仙石美, 五十嵐ひ, 町田雄. 東日本大震災における東北大学病院地域医療連携センターの活動 : 退院・転院調整および身元確認への支援等. 看護実践の科学 2012 06;37(6):46.

(33) 伊勢秀, 関口淳, 鈴木寛. インタビュートップが語る当日の行動と被害の実際 (その時トップはどう動いたか東日本大震災). Nikkei healthcare 2011 05(259):26.

(34) 伊澤敏. 東日本大震災--DMATから亜急性期の医療支援チーム派遣に関わって (特集東日本大震災における被災者支援の現状と教訓--現場からの報告と提言). The Japanese journal of quality and safety in healthcare 2011;6(2):235.

(35) 佐久間啓. Mental healthcare of refugees in the East Japan Earthquake Disaster. Journal of Japanese Association of Psychiatric Hospitals 2011 10;30(10):986.

(36) 佐々木隆. 東日本大震災の津波被災地における災害医療 (特集民医運の医師ここにあり!). 民医連医療 2011 09(469):14.

(37) 佐々木隆, 郷古親, 矢島剛. Verification of the medical services in the tsunami stricken area of the Great East Japan Earthquake : First response system of a disaster base hospital. 日本集団災害医学会誌 2012 07;17(1):9.

(38) 佐藤大, 阿部喜, 鈴木忠. "Expected" and "unexpected" in the disaster control headquarter of Tohoku University Hospital. 日本集団災害医学会誌 2012 07;17(1):21.

(39) 佐野博. From the Experience of the Great East Japan Earthquake : Did We Provide a Good Medical Service? 臨床整形外科 2012 03;47(3):211.

(40) 保田知. Precautions in providing medical treatments beyond physician's own specialty during emergency medical relief activities and in medical care at evacuation shelters. The Japanese journal of quality and safety in healthcare 2011;6(2):252.

(41) 内山巌. Chronic health effects of inhalation of dust or sludge. 日本医師会雑誌 2012 04;141(1):61.

(42) 内田貴, 深津亮. A report on disaster relief and medical support from Kesennuma, Miyagi prefecture. 老年精神医学雑誌 2012 02;23(2):185.

(43) 冨山陽. 震災発生時の医療機関におけるリハビリテーション部門の活動 (特集東日本大震災と理学療法). 理学療法ジャーナル 2012 03;46(3):191.

(44) 冨山陽. 東日本大震災に際して(4)被災地のリハビリテーション : 東日本大震災で損傷を免れた病院として : 宮城県. The Japanese journal of rehabilitation medicine 2011 12;48(12):769.

(45) 冲永壯. Reports from the disaster area: Major medical issues in the disastered elderly by M-9 earthquake on March 11, 2011. Japanese journal of geriatrics 2011 09;48(5):485.

(46) 出光俊. 岩手県大船渡市における被災地医療支援 : 避難所巡回診療を終えて (東日本大震災と皮膚科診療). 日本臨床皮膚科医会雑誌 2011 11/15;28(6):816.

(47) 出口宝, 富田秀, 近藤豊. An Evaluation of Medical Disaster Relief Services Provided to Survivors Following the 2011 Japanese Earthquake and Tsunami and during Initial Reconstruction. 日本医師会雑誌 2012 02;140(11):2361.

(48) 前田佐. 東日本大震災と精神科病院--被災地における支援者の心のケア (特集東日本大震災と精神科病院). Journal of Japanese Association of Psychiatric Hospitals 2011 10;30(10):996.

(49) 前田省, 山本裕. 医療ニーズの把握と精神的援助 (東日本大震災の経験を共有する) -- (避難所巡回での実践). The Japanese journal of nursing arts 2011 10;57(12):1129.

(50) 加藤圭. Measures of Local Ophthalmologists Association. 日本コンタクトレンズ学会誌 2011;53(4):302.

(51) 加藤寛, 鈴木友, 金吉. 座談会自然災害後の精神保健医療の対応について (特集東日本大震災(1)). トラウマティック・ストレス : 日本トラウマティック・ストレス学会誌 2011;9(2):152.

(52) 加賀谷健, 木ノ内聡, 奥山希. 宮城県女川地区歯科医療救護派遣報告 : 第8陣 (口腔病学会特別例会講演抄録東日本大震災に関する歯科保健医療支援活動). 口腔病学会雑誌 2012 03;79(1):39.

(53) 勝見敦, 丸山嘉, 内藤万. Medical relief activities of Japanese red cross society in The Great East Japan Earthquake. 日本集団災害医学会誌 2012 07;17(1):108.

(54) 原崇, 青木雅, 石渡勇. 東日本大震災の県内産婦人科医療施設への影響と復旧の状況 (特集東日本大震災と周産期) -- (発生直後の状況,経時的な改善状況). 周産期医学 2012 03;42(3):319.

(55) 原徳. 「語ろう!聞こう!会」からみえたこと : 松江赤十字病院の派遣者のこころのケア (特集東日本大震災から1年今後に活かす災害支援). 看護管理 2012 03;22(3):195.

(56) 原田奈. アメリカから災害支援活動に参加して--NPコース修了者から見た現場 (特集東日本大震災への医療支援の記録--日本赤十字社の取り組みと被災地からの報告). Japanese journal of nursing administration 2011 07;21(8):687.

(57) 又木満. 看護師としての支援ライフラインの寸断された女川で (緊急特集東日本大震災). Monthly community medicine 2011 05;25(5):444.

(58) 及川史. 震災当日の看護活動を振り返って(東日本大震災支援プロジェクト報告1). journal of Japan Academy of Gerontological Nursing 2012 03/31;16(2):95.

(59) 及川忠. Support activities provided by rehabilitation hospital after the Great East Japan Earthquake. 総合リハビリテーション 2012 03;40(3):227.

(60) 及川隆, 松坂薫, 近江谷留. Management of Bedridden Patients during the Higashi-Nihon Daishinsai Earthquake in Hachinohe National Hospital. 医療 : 国立医療学会誌 2012 05;66(5):197.

(61) 古川宗, 久志本成, 山内聡. 東日本大震災におけるDMATの役割について (特集救命救急医療 : その役割と問題点). Cefiro : 最新医療情報誌 2011(14):16.

(62) 古西勇, Konishi I. 新潟県内避難所での理学療法士としての関わり（特集 : 東日本大震災）. 新潟医療福祉学会誌 2011 12;11(2):12.

(63) 吉原克, 横室浩, 田巻一. The early medical dispatching against the Great East Japan Earthquake: the experience of Toho University Medical Centers. 東邦医学会雑誌 2011 05;58(3):158.

(64) 吉田孝. 災害時のプライマリ・ケア医の役割--東日本大震災における福島県での医療支援の経験から (特集東日本大震災における被災者支援の現状と教訓--現場からの報告と提言). The Japanese journal of quality and safety in healthcare 2011;6(2):242.

(65) 吉田索, 飯沼泰, 平山裕. 東日本大震災における周産期医療の経験 : 母児ともに緊急治療を必要とした症例を通して. 周産期医学 2012 03;42(3):393.

(66) 和田努. 医療--新たな胎動(第66回)"災害支援ナース"への期待--東日本大震災での看護師の活動. 健康保険 2011 10;65(10):46.

(67) 國光文, 岡田千, 道上幸. 東日本大震災における国立病院機構の医療支援活動. 公衆衛生 2012 02;76(2):138.

(68) 坂本レ, 楢舘恵, 北村京. 透析室における地震災害アクションカードの作成--震度6弱の地震シミュレーションを通して (特集東日本大震災--初期から今に至る災害医療活動からみえてきた課題). The Japanese journal of clinical nursing,monthly 2011 11;37(13):1814.

(69) 坂本一. Of the welfare system university student Attitude survey of the East Japan great earthquake disaster : Knowledge and grope for the uneasiness of the earthquake. 福岡医療福祉大学紀要 2012(9):93.

(70) 城川雅, 中野智, 落合紀. Medical evacuation after a major earthquake in Tokyo Metropolis using transient medical evacuation shelters experiences gained during the Great East Japan Earthquake. 日本集団災害医学会誌 2012 07;17(1):234.

(71) 城戸口和. 石巻赤十字病院における黄色治療班での活動 (東日本大震災の経験を共有する) -- (被災地での実践). The Japanese journal of nursing arts 2011 10;57(12):1085.

(72) 堀内龍, 安岡俊. The role of pharmacists in the emergency assistance system on the great disaster with big earthquake and tsunami. 日本医師会雑誌 2012 04;141(1):45.

(73) 大内啓, 渡瀬剛, 永松聡. Lets start! 災害医療(第35回)東日本大震災から学ぶこと(2)海外から見た東日本大震災 : 海外からの医療支援. 救急医療ジャーナル : 救急医療専門情報誌 2011 12;19(6):60.

(74) 大友康. 救急医療の今がわかる! EMERGENCY TOPIC 東日本大震災におけるDMAT活動 : DMAT活動の課題と今後の活動の展開. Emergency care 2012 03;25(3):301.

(75) 大塚尚, 山下亜, 木村慶. Experience of DMAT Rescue Activity by Doctor-helicopter in Tohoku Area after the Earthquake. 麻酔 2012 07;61(7):771.

(76) 大塚耕, 酒井明. The situation and psychiatric care of Iwate prefecture about the Great East Japan Earthquake : Psychiatric care to elderly people. 老年精神医学雑誌 2012 02;23(2):155.

(77) 大庭正. What was feasible and infeasible in the medical care activities of the Miyagi Prefectural Disaster Countermeasure Headquarters after the Great East Japan Earthquake? : Securing of communication means via MCA radio and roles of disaster medical care coordinators. 日本集団災害医学会誌 2012 07;17(1):130.

(78) 大木一, 栗原聰, 鈴木光, 久保田裕, 松尾康. 前橋赤十字病院における東日本大震災に対する災害救援について. The Kitakanto medical journal 2011 11/01;61(4):555.

(79) 大林由. 石巻赤十字病院に肋産師,看護師,ER支援要員を派遣--大災害時の施設支援要員派遣体制の確立が求められている (特集東日本大震災への医療支援の記録--日本赤十字社の取り組みと被災地からの報告). Japanese journal of nursing administration 2011 07;21(8):627.

(80) 大野か. 東日本大震災での避難所における初期保健活動の実際 (特集東日本大震災--初期から今に至る災害医療活動からみえてきた課題). The Japanese journal of clinical nursing,monthly 2011 11;37(13):1794.

(81) 天野教. 開業医の支援チームができること--JMATとして宮城県女川町に出動して (東日本大震災--いま医療にできること). Japan medical journal 2011 04/09(4537):32.

(82) 太田晴, 中村惠. The action report for the Great East Japan Earthquake : Connected supporting action to JMAT and Public health center. 日本集団災害医学会誌 2012 07;17(1):273.

(83) 奥村順. 被災地における公衆衛生活動--東日本大震災の経験から (特集これからの災害医療に向けて) -- (災害サイクルに応じた薬剤師業務のポイント). The Pharmaceuticals monthly 2011 09;53(9):1317.

(84) 安東由. 医療支援・石巻での3日間--東日本大震災・思い出すままに. Schneller 2011(79):43.

(85) 安東由. Inquiry about the March 11th, 2011 disaster in Eastern Japan (4) Problems in disaster: focusing on chronic disorders. 臨床検査 2011 10;55(10):1024.

(86) 安田俊. 出張先の病院で震災に遭遇して東日本大震災の体験 (緊急特集東日本大震災). Monthly community medicine 2011 05;25(5):432.

(87) 宮崎国. 第1陣として被災地入りして東日本大震災災害支援について (緊急特集東日本大震災). Monthly community medicine 2011 05;25(5):441.

(88) 宮崎隆. Feature articles: Staff of department of dentistry and postgraduate's activity reports in Showa University medical treatment rescue corps in East Japan Great Earthquake. Dental medicine research 2011 07/31;31(2):185.

(89) 宮脇博. Medical Supportive Activities of the DMAT of the National Defense Medical College in the Great East Japan Earthquake. 人間工学 : 日本人間工学会誌 2012;48(3):127.

(90) 寺田宙, 鈴木晃, 秋葉道. Environmental health : A study based on the earthquake disaster. 保健医療科学 2011 12;60(6):484.

(91) 小井土雄, 近藤久, 市原正. 東日本大震災における災害派遣医療チーム(DMAT)の活動と課題 (特集災害医療と被害管理). 医薬ジャーナル 2012 02;48(2):686.

(92) 小井土雄, 近藤久, 市原正. 東日本大震災におけるDMAT活動と課題 (特集病院と日本復興). 病院 2012 01;71(1):48.

(93) 小原聡, 長橋美, 横野富. 宮城県,精神保健医療福祉の現状と課題 (特集東日本大震災から6か月,被災地支援の今後) -- (インタビュー東北三県から見る,被災地支援のこれまでと今後). The Japanese journal of psychiatric nursing 2011 10;38(10):25.

(94) 小林道, 小林正, 石橋悟. The long-term and large-scale patient transportation from our hospital in the Great East Japan Earthquake : For the wide-area medical transportation and for maintenance of hospital function. 日本集団災害医学会誌 2012 07;17(1):99.

(95) 小笠原敏. 被災地からのレポート東日本大震災その時,被災地にある岩手県立大船渡病院産婦人科では(3・最終回)未来に向かって--妊婦見守りネットワークと震災に強いネットワークの構築. The Japanese journal for midwives 2011 09;65(9):820.

(96) 小笠原敏. 被災地からのレポート東日本大震災その時,被災地にある岩手県立大船渡病院産婦人科では(2)妊婦を守る--岩手県大船渡病院産婦人科のポリシー. The Japanese journal for midwives 2011 08;65(8):718.

(97) 小笠原敏. 被災地からのレポート東日本大震災その時,被災地にある岩手県立大船渡病院産婦人科では(1)大震災から7日間の出来事--何も考えることができず,とても苦しかった7日間. The Japanese journal for midwives 2011 07;65(7):598.

(98) 小賀坂奈, 佐藤め, 宮崎博. Activities as DMAT of the disaster core hospital in the Great East Japan Earthquake at Fukushima Medical University Hospital. 日本集団災害医学会誌 2012 07;17(1):66.

(99) 小越佐. 全国17関連校から市内唯一の石巻赤十字看護専門学校を支援--泥を削り落としながら流出した書類を復元 (特集東日本大震災への医療支援の記録--日本赤十字社の取り組みと被災地からの報告). Japanese journal of nursing administration 2011 07;21(8):672.

(100) 小野寺直, 櫻井滋. 東日本大震災--薬剤師はどう動いたか(第5回)被災地における薬剤師の医療支援および感染対策活動. The Pharmaceuticals monthly 2011 10;53(11):1781.

(101) 尾形英. The Experience of Medical Support for Respiratory Disease in the Fundamental Hospital (Iwate Prefecture) of Disaster Area. 日本胸部臨床 2012 03;71(3):216.

(102) 山内広. 内科から : 東日本大震災・大津波被災時の呼吸器・アレルギー疾患診療の問題点 (特集東日本大震災とアレルギー疾患) -- (現地活動報告). アレルギー・免疫 2012 04;19(4):512.

(103) 山内広. Problems of home oxygen therapy in tsunami. 日本医師会雑誌 2012 04;141(1):50.

(104) 山内真, 三上純, 佐藤大. Actual state of medical care support for radiation victims at Advanced Critical Care and Emergency Center, Hirosaki University School of Medicine Hospital, after the Great East Japan Earthquake and Fukushima Nuclear Power Plant accident, and problems to be solved. 日本集団災害医学会誌 2012 07;17(1):160.

(105) 山内聡, 赤松順, 阿部喜. Preparations for Receiving DMATs at Operational Headquarters : Learning from Pre- and Post-earthquake Experience. 日本集団災害医学会誌 2011 10;16(2):231.

(106) 山城直. 東日本大震災の医療支援に参加して. 社会労働衛生 2011 05;8(3):28.

(107) 山岡ふ. 初動班は移動しながらより救護が必要な地域を探す--栗原市で情報収集し,気仙沼市を経て陸前高田市へ (特集東日本大震災への医療支援の記録--日本赤十字社の取り組みと被災地からの報告). Japanese journal of nursing administration 2011 07;21(8):636.

(108) 山崎學. 東日本大震災を体験して (特集東日本大震災と精神科病院). Journal of Japanese Association of Psychiatric Hospitals 2011 10;30(10):942.

(109) 山本保. インタビュー災害時における医療支援はニーズを見越しての提供・撤退が重要 (特集東日本大震災医療支援の教訓と復興への道程). MD 2011 06;8(6):24.

(110) 山田隆. 東日本大震災から考える、地域医療における医療健康情報 (特集クラウド利用へ向かう医療情報と危機管理). A Monthly journal of medical imaging and information 2011 07;43(7):618.

(111) 山野目辰. The Acute Stage Disaster Medical Action at Iwate Prefectural Ofunato Hospital and Chronic Stage Disaster Medical Management in Ofunato District in Tohoku Region Pacific Coast Earthquake and Tsunami. 日本集団災害医学会誌 2012 07;17(1):117.

(112) 山野目辰. The acute stage disaster medical action at Iwate Prefectural Ofunato Hospital in Tohoku region pacific coast earthquake and tsunami. 日本医師会雑誌 2012 04;141(1):19.

(113) 岡田史, Okada F. 災害時における介護課題の検討 : 日本介護福祉士会の東日本大震災介護支援ボランティア活動を中心として（特集 : 東日本大震災）. 新潟医療福祉学会誌 2011 12;11(2):55.

(114) 岩井志. 日本医師会災害医療チーム(JMAT)での活動と今後の課題 (緊急時の動きを学ぶ東日本大震災における手術室看護師の経験と今後に向けた対策(1)). OPE nursing : The Japanese journal of operating room nursing 2012 01;27(1):96.

(115) 岩崎鋼. 東日本大震災診療報告. 漢方の臨床 2011 05/25;58(5):1013.

(116) 岩田健. 医療従事者の知っておきたい 2011年の東日本大震災と感染対策. Infection control 2011 05;20(5):430.

(117) 川井朋. 東日本大震災被災地医療ボランティア活動の報告. The Japanese journal of acupuncture & manual therapies 2011 07;70(7):97.

(118) 川嶋隆, 吉田剛, 岡田直. The action report of Kobe University Hospital in the Great East Japan Earthquake. 日本集団災害医学会誌 2012 07;17(1):246.

(119) 川野健, 竹島正, 白神敬. A framework of suicide prevention and support reconstruction of community mental health. 精神保健研究 2012(25):35.

(120) 市川光. 小児災害救急医療の現状と課題 : 東日本大震災支援医療を経験して. The Journal of the Japan Pediatric Society 2011 08/01;115(8):1285.

(121) 市川光. 災害時の小児救急医療とその課題 : 日本小児救急医学会の東日本大震災支援医療活動を経験して (特集大災害と母子保健) -- (東日本大震災から学ぶ災害時の母子の保健と福祉). 母子保健情報2011 11(64):6.

(122) 市川幾. 東日本大震災災害医療救援隊派遣と看護部の対応そして看護職員メンタルヘルス支援看護管理者育成の視点(第5回)メンタルヘルスの支援. 師長主任業務実践 2011 05/01;16(338):11.

(123) 平井基. Disaster-response Medical Services Report : dental Support in Iwaki City and Minamisanriku Town. 歯科学報 2012 04;112(2):115.

(124) 廣川進. Critical incident stress and mental health care in the Japan Coast Guard: improvement in organizational treatment and measure. Journal of Japanese Association of Psychiatric Hospitals 2011 10;30(10):1002.

(125) 廣瀬輝. 東日本大震災への医療介護の対応. JMS 2011 06:5.

(126) 後藤え, 石井幹, 門間典. 被災地報告東日本大震災における東北大学病院の災害看護トリアージ対応を含めた外来機能における看護活動報告. 看護管理 2012 03;22(3):217.

(127) 成田徳. 3.11東日本大震災における医療コーディネーターの活動--気仙沼からの報告 (特集東日本大震災における被災者支援の現状と教訓--現場からの報告と提言). The Japanese journal of quality and safety in healthcare 2011;6(2):255.

(128) 新井順. 東日本大震災時の茨城県立こども病院新生児病棟 (特集東日本大震災と周産期) -- (発生直後の状況,経時的な改善状況). 周産期医学 2012 03;42(3):323.

(129) 有賀徹, 石川育, 石原哲. Disaster medicine : The instructive and controversial through Great East Japan Earthquake and Tsunami Disasters. 日本医師会雑誌 2012 04;141(1):5.

(130) 望月英, 水八, 若林進. 東日本大震災を契機にみえたMRの使命 : 情報伝達の重要性(PART1)災害医療現場で支援チームにMRが貢献. ミクス 2012 04;40(5):32.

(131) 朝田隆. Mental Support for the Victim of Japan Earthquake. 精神神経学雑誌 2012;114(3):227.

(132) 本村啓. 九州大学病院精神科の活動について (特集東日本大震災の支援から考える). ふくおか精神保健 2012 03(57):77.

(133) 村上典. 東日本大震災の被災者に対するこころのケア懸念される「複雑性悲嘆」に医療的ケアが必要 (特集見逃さない、再発させないプライマリ・ケアで診る「うつ病」). MD 2011 08;8(8):18.

(134) 村岡正. 避難先でも、医者だった--被災から怒濤の2日間の記録 (特集地震、津波…その時医療は--東日本大震災から現在まで). 月刊保団連 2011 10:4.

(135) 村川雅. Our experience of the 2011 Great East Japan Earthquake and subsequent Fukushima Daiichi nuclear power plant accident. 医学のあゆみ 2011 12/10;239(11):1107.

(136) 東泰. 被災地における地域中核病院の病棟での対応 (特集東日本大震災への医療支援の記録--日本赤十字社の取り組みと被災地からの報告). Japanese journal of nursing administration 2011 07;21(8):697.

(137) 松岡芳. 南相馬市立総合病院での皮膚科診療支援報告と震災時の皮膚科医の役割 (東日本大震災と皮膚科診療). 日本臨床皮膚科医会雑誌 2011 11/15;28(6):803.

(138) 松本和. Mental health care after the Great East Japan Earthquake. 日本医師会雑誌 2012 04;141(1):56.

(139) 松本昭, 前田順, 石田時. The report details my investigative research on effective infective infection counter measures at the time of disasters. 福島医学雑誌 2012 06;62(2):41.

(140) 松浦真. Physicians and disaster medical care : The changes in gastrointestinal bleeding and the countermeasures at a major hospital after the Great East Japan Earthquake and the Tsunami : Report from the hospital located in the disaster area. 日本内科学会雑誌 2012 08/10;101(8):2370.

(141) 柚原尚. 激甚災害地域における医療救護班としての活動報告 (特集東日本大震災--初期から今に至る災害医療活動からみえてきた課題). The Japanese journal of clinical nursing,monthly 2011 11;37(13):1804.

(142) 栗原正. 東日本大震災の被災者口腔ケア (特集災害医療). 難病と在宅ケア 2012 05;18(2):31.

(143) 榛沢和. 東日本大震災後における深部静脈血栓症(DVT)と問題点--新潟県中越地震の教訓を生かすには (特集東日本大震災における被災者支援の現状と教訓--現場からの報告と提言). The Japanese journal of quality and safety in healthcare 2011;6(2):248.

(144) 横室浩, 吉原克. Our initial disaster medical support of Toho University Omori Medical Center as a disaster medical hospital at Kesennuma-City. 東邦医学会雑誌 2011 05;58(3):167.

(145) 横田裕. Medical support for Great East Japan Earthquake in acute phase : The activities of Disaster Medical Assistance Team & Japan Medical Assistance Team. 日本医師会雑誌 2012 04;141(1):26.

(146) 横道弘. 被災病院の震災対策本部長として公立黒川病院の震災対応 (緊急特集東日本大震災). Monthly community medicine 2011 05;25(5):428.

(147) 樹神學, 佐藤宗, 高橋玄. 東日本大震災の被災地から(宮城県石巻市) (特集東日本大震災と精神科病院). Journal of Japanese Association of Psychiatric Hospitals 2011 10;30(10):963.

(148) 武田多. The Medical Support Team of Mie Prefecture to support reconstruction of the local medical system destroyed by the Great East Japan Earthquake. 日本集団災害医学会誌 2012 07;17(1):281.

(149) 武藤浩. 女川町立病院における薬剤師の活動--医薬品情報を災害医療に活かす取り組み (東日本大震災--薬剤師はどう動いたか(第6回)). The Pharmaceuticals monthly 2011 11;53(12):1939.

(150) 水谷幸. 東日本大震災と患者・障害者 (特集地震、津波…その時医療は--東日本大震災から現在まで). 月刊保団連 2011 10:27.

(151) 江畑俊. 日本皮膚科学会巡回医療チーム活動報告第5陣に参加して (東日本大震災と皮膚科診療). 日本臨床皮膚科医会雑誌 2011 11/15;28(6):800.

(152) 池上正. 東日本大震災現地取材石巻赤十字病院が遭遇した未曾有の災害医療災害看護. 看護ジャーナル 2011 05;1(1):12.

(153) 池田由. 石巻日赤・石巻看専支援要員に対するオリエンテーションの課題 (特集東日本大震災への医療支援の記録--日本赤十字社の取り組みと被災地からの報告). Japanese journal of nursing administration 2011 07;21(8):632.

(154) 池田由. 広域大災害における情報共有の実際と課題 (特集東日本大震災への医療支援の記録--日本赤十字社の取り組みと被災地からの報告). Japanese journal of nursing administration 2011 07;21(8):623.

(155) 泉眞, 中村邦, 近藤倫. 被災地における医療・介護--東日本大震災後の現状と課題. Issue brief 2011 06/02(713):1.

(156) 津田雅. 岩手県立釜石病院におけるDMAT活動 (東日本大震災の経験を共有する) -- (被災地での実践). The Japanese journal of nursing arts 2011 10;57(12):1093.

(157) 浅井康, 野村和, 佐藤昌. The Great East Japan Earthquake and medical disaster management. 放射線防護医療 2011 12(7):9.

(158) 浅野祥, 布施至, 櫻井淑, 田村正. 東日本大震災被災地からの活動報告. 日本小児科学会雑誌 2011 05/01;115(5):967.

(159) 深澤舞. NCNP Website to Provide Information of Disaster Mental Health. トラウマティック・ストレス : 日本トラウマティック・ストレス学会誌 2011;9(2):148.

(160) 渡辺洋. 東日本大震災と精神科医療 (東日本大震災--いま医療にできること). Japan medical journal 2011 06/18(4547):26.

(161) 渡邉暁, 西脇龍, 福田恵. 東日本大震災支援活動報告--石巻医療支援活動(2011年]3月21日〜3月28日) (薬剤師の被災地支援活動と医薬情報). Pharmaceutical library bulletin 2011;56(4):304.

(162) 渡邉美. 発災3日目,冠水が続くなか24時間体制の救護活動を支援 (特集東日本大震災への医療支援の記録--日本赤十字社の取り組みと被災地からの報告). Japanese journal of nursing administration 2011 07;21(8):643.

(163) 渡邉要. 医療救護班における理学療法・士の支援活動 (特集東日本大震災と理学療法). 理学療法ジャーナル 2012 03;46(3):221.

(164) 渡邊一, 高橋千, 松浦誠. The activity report of the intensive care unit of a disaster base hospital in the Great East Japan Earthquake stricken area : From the viewpoint of nursing administration. 日本集団災害医学会誌 2012 07;17(1):27.

(165) 渡部英. Medical information systems risk management to learn from the Japanese earthquake. 月刊新医療 2011 07;38(7):71.

(166) 濱崎允. 山形済生病院 (東日本大震災・本会済生会]被災地施設からの報告空前の困難、使命感は高まっていった). 済生 2011 06;87(6):48.

(167) 片山尚, 山野邊秀. 東日本大震災支援レポート. JMC 2011 06;19(2):88.

(168) 田中千. 日本医労連の緊急対策その時、医療労働者は…… (特集東日本大震災への医労連の取り組み--現状報告とこれからについて). 医療労働 2011 05(535):3.

(169) 田中博. Healthcare IT system in the midst of and after Great East Japan Earthquake Disaster : Grand design for reconstruction of Tohoku-region healthcare IT system. 情報管理 2012 03;54(12):825.

(170) 田中嘉. The Actual Condition of Medical Treatment and Care Management. 緩和ケア 2012 01;22(1):21.

(171) 田巻一, 一林亮, 宮崎め. Report on the DMAT response to the 2011 Great East Japan Earthquake. 東邦医学会雑誌 2011 05;58(3):162.

(172) 田村佳. 東日本大震災本当の復興の日を目指して被災地で管理栄養士ができること、しなければならないこと(第7回)病院で結成したチームによる避難所での活動. Nutrition care 2012 01;5(1):94.

(173) 田村佳. 東日本大震災本当の復興の日を目指して(新連載・第1回)被災地で管理栄養士ができること、しなければならないこと未曾有の大震災発生! そのとき病院は……. Nutrition care 2011 07;4(7):762.

(174) 甲斐聡, 村井隆, 松尾信. Analysis of medical coordination on accepting foreign medical team : Lessons learned from medical coordination of Israeli medical delegation on the Great East Japan Earthquake and Tsunami Disaster. 日本集団災害医学会誌 2012 07;17(1):214.

(175) 矢内勝. 研究会/施設紹介インタビュー The Front Line 東日本大震災とCOPD診療 : 災害医療における慢性期医療の重要性. International review of asthma & COPD 2012 07;14(2):89.

(176) 矢内勝. Respiratory Diseases after the Great East Japan Earthquake at a Designated Disaster Hospital in Miyagi Prefecture. 日本胸部臨床 2012 03;71(3):206.

(177) 矢田部裕, 藤瀬昇, 池田学. A report on disaster relief and medical support from Minamisanriku, Miyagi prefecture. 老年精神医学雑誌 2012 02;23(2):181.

(178) 知覧俊. 医療トレンド(第4回)東日本大震災の感染症対策. Nursing today 2011 08;26(4):56.

(179) 石井哲. Medical support for the victims of Great East Japan Earthquake : What we could and what we couldn't? : An experience of participating in Japan Medical Association Team. 広島医学 2012 02;65(2):106.

(180) 石井幹, 後藤え, 門間典. 被災地報告東日本大震災における東北大学病院の災害看護看護管理室の活動報告. 看護管理 2012 03;22(3):211.

(181) 石井正. The Report of the Great East Japan Earthquake relief activities in the Ishinomaki medical district : From the perspective of a Miyagi Prefecture disaster medical care coordinator. 日本集団災害医学会誌 2012 07;17(1):92.

(182) 石井正. 東日本大震災に対する災害救護活動 (東日本大震災と災害医療). 日本病院会雑誌 2011 10;58(10):1103.

(183) 石井正. The role of JMAT as a response for a disaster of Japan Medical Association. 日本医師会雑誌 2012 04;141(1):32.

(184) 石井正. 東日本大震災における対応 : JMAT:日本医師会災害医療チーム (特集病院と日本復興). 病院 2012 01;71(1):53.

(185) 石井正. JMATの活動と、東日本大震災における課題 (特集地震、津波…その時医療は--東日本大震災から現在まで). 月刊保団連 2011 10:33.

(186) 石井美. 災害時に必要な看護とは--東日本大震災での災害支援ナースの活動から (特集東日本大震災における被災者支援の現状と教訓--現場からの報告と提言). The Japanese journal of quality and safety in healthcare 2011;6(2):258.

(187) 石橋悟, 小林道, 小林正. Acute phase medication after the Grate East Japan Earthquake. 日本集団災害医学会誌 2012 07;17(1):32.

(188) 穏土ち. 東日本大震災と障害者の医療・介護について. 介護保険情報 2011 07;12(4):18.

(189) 笹岡眞. 災害ソーシャルワーク--東日本大震災への医療ソーシャルワーカーの取り組み (特集緊急報告、震災支援の現状と課題). 地域ケアリング 2011 07;13(7):17.

(190) 緑川浩. 「東日本大震災」に遭遇して (特集大震災を超えて進もう介護と医療). Senior community 2011 05:14.

(191) 臼井千. 発災初期の実践活動からみえてきた課題 (特集東日本大震災--初期から今に至る災害医療活動からみえてきた課題). The Japanese journal of clinical nursing,monthly 2011 11;37(13):1788.

(192) 船橋香. 災害支援の経験から--避難所での支援と災害支援派遣体制について考える (特集東日本大震災--初期から今に至る災害医療活動からみえてきた課題). The Japanese journal of clinical nursing,monthly 2011 11;37(13):1811.

(193) 荒井陽, 里見進. 東日本大震災--被災地医療の現況と東北大学病院の取り組み (特集東日本大震災と日本泌尿器科学会). The Japanese journal of urology 2011 07;102(4):7.

(194) 荒巻東. 派遣肋産師として,業務変更に対応しながら急増した分娩を介助した (特集東日本大震災への医療支援の記録--日本赤十字社の取り組みと被災地からの報告). Japanese journal of nursing administration 2011 07;21(8):664.

(195) 葛西龍. 被災の現場から家庭医が語る (特集東日本大震災を経て再考するいま本当に必要な医療のものさしとは何か). Konica Minolta medical network 2011;62(2):10.

(196) 藍原寛. 全国のJMATを中心とした支援で避難所での死亡者がゼロに--いわき市医師会木田光一会長に聞く (特集東日本大震災への医療支援の記録--日本赤十字社の取り組みと被災地からの報告). Japanese journal of nursing administration 2011 07;21(8):692.

(197) 藤原一. 川俣病院 (東日本大震災・本会済生会]被災地施設からの報告空前の困難、使命感は高まっていった). 済生 2011 06;87(6):60.

(198) 藤原幾. 大規模災害時における糖尿病をもつ子どもの医療 (特集小児における糖尿病看護 : 糖尿病をもつ子どもの成長発達に沿った看護をめざして) -- (糖尿病をもつ子どもを取り巻く社会状況の変化). 小児看護 2012 02;35(2):209.

(199) 藤森敬, 野村泰, 安田俊. 福島県産科 : 震災直後の産科医療と妊娠動向 (特集東日本大震災と周産期) -- (発生直後の状況,経時的な改善状況). 周産期医学 2012 03;42(3):303.

(200) 西村司. 東日本大震災でのNPO健康運動指導士会東北各支部の活動調査報告. 体力科學 2012 02/01;61(1):13.

(201) 西田伸. 石巻市での医療救護活動の経験と今後の教訓 (特集東日本大震災の教訓). 難病と在宅ケア 2011 09;17(6):21.

(202) 諸江雄, 二宮宣, 久野将. What is the "SAFETY" on confined space medicine (CSM)? : Learning from an experienced "CSM" in the crash spot at a parking slope of the supermarket in Tokyo by the Great East Japan Earthquake. 日本集団災害医学会誌 2012 07;17(1):45.

(203) 賀来進. 宮城県南三陸町に歯科医療支援に入って (東日本大震災記録集--未曾有の災害を乗り越えて) -- (支援活動の手記). 月刊保団連 2011 09:40.

(204) 越智文. Medical Assistance provided by the Japan Self Defense Force after the Great East Japan Earthquake. The Japanese journal of rehabilitation medicine 2011 12;48(12):779.

(205) 足立了, 田中義. 東日本大震災--いま医療にできること大規模災害時の肺炎予防--口腔保健の重要性. Japan medical journal 2011 04/16(4538):29.

(206) 辰濃哲, 今岡洋. 東日本大震災医療気仙沼市立病院ドキュメント脇役が支えた災害医療. Aera 2011 04/18;24(18):27.

(207) 遠藤史, 徳田浩, 八田益. Outbreak of Influenza A at the Refuge Center after the East Japan Great Earthquake Disaster. 日本環境感染学会誌 2012;27(1):50.

(208) 遠藤智. 避難者支援の経過--こころのケアチームの支援活動を通して (特集東日本大震災と精神科病院). Journal of Japanese Association of Psychiatric Hospitals 2011 10;30(10):991.

(209) 遠藤秀. 被災した県立釜石病院から東日本大震災報告 (緊急特集東日本大震災). Monthly community medicine 2011 05;25(5):422.

(210) 酒井明. 東日本大震災急性期における高齢者の健康問題が及ぼす影響と看護. 老年医学=Geriatric　Medicine 2012 03;50:309.

(211) 野口三, 渡邉亮, 高橋秀. At the Great East Japan Earthquake on March 11, the Eye Medical Activity in Ishinomaki Red Cross Hospital. 日本コンタクトレンズ学会誌 2011;53(4):286.

(212) 野澤正. 東日本大震災--いま医療にできること DMAT活動の報告--いち小児科医が思うこと. Japan medical journal 2011 06/04(4545):27.

(213) 野田三. 2カ月間の被災地・避難所における看護活動報告--被災者とともにあることとは (特集東日本大震災--初期から今に至る災害医療活動からみえてきた課題). The Japanese journal of clinical nursing,monthly 2011 11;37(13):1796.

(214) 金吉, 秋山剛, 大沼麻. Initial mental health response after the 311 Great Eastern Earthquake in Japan. 精神保健研究 2012(25):15.

(215) 金子健. 震災前の6倍の患者が集中する石巻赤十字病院でER支援活動 (特集東日本大震災への医療支援の記録--日本赤十字社の取り組みと被災地からの報告). Japanese journal of nursing administration 2011 07;21(8):669.

(216) 鈴木友. Community Mental health service after the Great East Japan Earthquake. 精神保健研究 2012(25):21.

(217) 鈴木友. Mental health service after the Great Eastern Japan Earthquake. Journal of Japanese Association of Psychiatric Hospitals 2011 10;30(10):957.

(218) 鈴木正. 災害支援活動(6)東日本大震災を経験して菅間記念病院の初期対応と災害被災者の受け入れ. 師長主任業務実践 2011 08/01;16(344):36.

(219) 長岡信. 東日本大震災緊急災害支援レポート災害医療の現状と対応策災害急性期における整形外科領域の医療. 整形外科surgical technique : 手術が見える・わかる専門誌 2011;1(3):358.

(220) 長瀬文. 東日本大震災・原発事故にどのように立ち向かったか--すべての被災者に寄り添い、多くの人々と心ひとつに (特集オール民医連で震災に立ち向かおう). 民医連医療 2011 06(466):6.

(221) 門間典, 後藤え, 石井幹. 3.11東日本大震災東日本大震災における大学病院災害看護--とっさに行なわれた看護行為に焦点を当てて. The Japanese journal of nursing science 2011 11;36(12):37.

(222) 間嶋健. Consultation support of medical social workers for at-home users of artificial respirators following the Great East Japan Earthquake : Through the operations of the emergency consultation center for at-home medical patient users of artificial respirators. 日本集団災害医学会誌 2012 07;17(1):196.

(223) 関根忠, 菊地健. 一般病院を軸にした震災後の理学療法・士の支援活動 (特集東日本大震災と理学療法). 理学療法ジャーナル 2012 03;46(3):209.

(224) 阿南英. Relation of DMAT activity and the medical disease in the East Japan great earthquake disaster. 日本内科学会雑誌 2012 04/10;101(4):1132.

(225) 雪田慎. 被災地の自治体職員と医療保健福祉関係者のメンタルヘルスの在り方について--岩手、福島、宮城での現地調査や相談活動などの体験を通して (特集東日本大震災の復旧・復興作業からいのちと健康を守るために). 働くもののいのちと健康 2011:9.

(226) 青木正. 東日本大震災の医療現場とその教訓 (特集東日本大震災の教訓). 難病と在宅ケア 2011 09;17(6):8.

(227) 飛鳥井望. Acute stress disorder and posttraumatic stress disorder. Journal of Japanese Association of Psychiatric Hospitals 2011 10;30(10):945.

(228) 飯島勝. Disaster medicine in the elderly evacuees : What should we learn from 2011 Tohoku earthquake? 日本老年医学会雑誌 2012 03;49(2):164.

(229) 香山明, 酒井道, 神山利. 東日本大震災から,私たちは何をしてきたのか : 精神科病院に勤務する作業療法士からの報告 (特集障害のある方,要介護高齢者を災害時に支える). 地域リハビリテーション 2012 02;7(2):125.

(230) 高山順. 東日本大震災への医療支援--長野県医師会医療救護班として被災地へ (東日本大震災に寄せて(2)) -- (被災地支援で私たちが見たもの・感じたこと). The Japanese journal of psychiatric nursing 2011 06;38(6):73.

(231) 高橋宏. 東日本大震災 : その時医療現場は(その2)茨城県基幹病院手術室の被害状況アンケート調査 (特集緊急レポート). 日本手術医学会誌 2012 05;33(2):122.

(232) 高橋寛, 小松真, 安田哲. 東日本大震災--薬剤師はどう動いたか(第4回)現地での取り組みと医療支援を通じて感じたこと. The Pharmaceuticals monthly 2011 09;53(9):1331.

(233) 高橋敏. 東日本大震災医療救援活動に参加して (特集東日本大震災に派遣された昭和大学医療救援隊における歯学部職員・大学院生の活動報告). Dental medicine research 2011 07;31(2):193.

(234) 高橋智. The disaster medicine and dementia care after the Great East Japan Earthquake in Iwate prefecture. 老年精神医学雑誌 2012 02;23(2):150.

(235) 高橋浩. 東日本大震災の災害医療に参加して--岩手県山田町における昭和大学医療救援隊の歯科医療活動について (特集東日本大震災に派遣された昭和大学医療救援隊における歯学部職員・大学院生の活動報告). Dental medicine research 2011 07;31(2):186.

(236) 高橋秀, 長尾圭, 神尾陽. Child and adolescent psychiatric support in the Great East Japan Earthquake. 精神保健研究 2012(25):43.

(237) 高橋秀. 眼科から : 拠点病院での眼科医として東日本大震災を体験して (特集東日本大震災とアレルギー疾患) -- (現地活動報告). アレルギー・免疫 2012 04;19(4):540.

(238) 高田勝, 原田真. 東日本大震災--薬剤師はどう動いたか(第3回)日本赤十字社医療救護班として. The Pharmaceuticals monthly 2011 08;53(8):1175.

(239) 高里良. 東日本大震災を振り返り病院災害医療を考える. 日本病院会雑誌 = Journal of Japan Hospital Association 2011 09/01;58(9):968.

(240) 高階憲. The day the Great East Japan Earthquake and tsunami attacked our hospital. 精神科 2011 12;19(6):577.

(241) 黒木一, 倉恒正, 阿部伸. Report on activities of the DMAT of JA Hiroshima General Hospital after the earthquake in northeastern Japan. The journal of the Hiroshima Medical Association 2011 05;64(5):256.

(242) 黒田裕. 地域の人々のこころを支える医療 : 東日本大震災の在宅医療の現場から (特集地域のいのちを守るために : 医療崩壊を乗り越える). ガバナンス 2011 12(128):30.

(243) 黒田裕. 災害看護ボランティア活動の拠点として (特集東日本大震災--初期から今に至る災害医療活動からみえてきた課題). The Japanese journal of clinical nursing,monthly 2011 11;37(13):1790.

(244) 齊藤修. 救急医療の今がわかる! EMERGENCY TOPIC 東日本大震災における小児診療の経験から : 日本小児救急医学会医療支援隊活動からの報告. Emergency care 2012 04;25(4):404.

(245) 齋藤充. 被災地から女川より (緊急特集東日本大震災). Monthly community medicine 2011 05;25(5):417.

III. Books written in Japanese

(1) 日経ドラッグインフォメーション． 東日本大震災取材班ドキュメント東日本大震災 そのとき薬剤師は医療チームの要になった．日経BP社: 2011/06

(2) 辰濃哲郎, 医薬経済編集部．ドキュメント・東日本大震災 「脇役」たちがつないだ震災医療 ．医薬経済社: 2011/06

(3) 久志本 成樹．石巻赤十字病院、気仙沼市立病院、東北大学病院が救った命．アスペクト： 2011/08

(4) 海堂 尊 (監修)．救命―東日本大震災、医師たちの奮闘．新潮社: 2011/08

(5) 由井 りょう子, 石巻赤十字病院．石巻赤十字病院の100日間．小学館: 2011/09

(6) 日本看護協会出版会編集部 (編集)．ナース発東日本大震災レポート―ルポ・そのとき看護は．日本看護協会出版会 : 2011/09

(7) 中原一歩．奇跡の災害ボランティア「石巻モデル」．朝日新聞出版: 2011/10

(8) 山崎 達枝．3.11東日本大震災 看護管理者の判断と行動．日総研出版: 2011/10

(9) 菅波茂．AMDA被災地へ!―東日本大震災国際緊急医療NGOの活動記録と提言．小学館スクウェア: 2011/12

(10) 京都民医連中央病院東日本大震災支援対策本部 (編集)．災害支援と地域づくり―東日本大震災の支援活動の記録 暮らしに生きる学問をめざす．せせらぎ出版: 2011/12

(11) 西澤匡史, 杉本勝彦．いのちを守る―東日本大震災・南三陸町における医療の記録．へるす出版: 2012/02

(12) 石井 正．東日本大震災 石巻災害医療の全記録―「最大被災地」を医療崩壊から救った医師の7カ月．講談社 : 2012/02

(13) 上田 耕蔵．東日本大震災、医療と介護に何が起こったのか―震災関連死を減らすために．萌文社: 2012/07
